# Modulation of platelet-derived microparticles to adhesion and motility of human rheumatoid arthritis fibroblast-like synoviocytes

**DOI:** 10.1371/journal.pone.0181003

**Published:** 2017-07-13

**Authors:** Wenwen Wang, Jiahuan Liu, Binzhou Yang, Zhongshuang Ma, Guiping Liu, Weigan Shen, Yu Zhang

**Affiliations:** 1 Clinical Medical College, Yangzhou University, Yangzhou, P.R. China; 2 School of Medicine, Yangzhou University, Yangzhou, P.R. China; 3 The Third People’s Hospital of Chengdu, Southwest JiaoTong University College of Medicine, Chengdu, P.R. China; 4 Department of Rheumatology, Yancheng Chengnan Hospital, Yancheng, P.R. China; 5 Jiangsu Key Laboratory of Integrated Traditional Chinese and Western Medicine for Prevention and Treatment of Senile Diseases, Yangzhou, P.R. China; 6 Jiangsu Co-innovation Center for Prevention and Control of Important Animal Infectious Diseases and Zoonoses, Yangzhou, P.R. China; Seoul National University College of Pharmacy, REPUBLIC OF KOREA

## Abstract

Platelet-derived microparticles (PMPs) are closely associated with disease activity in rheumatoid arthritis (RA) and contribute to the inflammatory process. Rheumatoid arthritis fibroblast-like synoviocytes (RA-FLSs) play important roles in the progression of joint destruction. The aim of this study is to demonstrate whether PMPs affect the adhesion and motility of RA-FLSs. Our data indicated that PMPs promoted migration, invasion and adhesion to extracellular matrix (ECM) of RA-FLSs. Further study showed that PMPs up-regulated the expression of matrix metalloproteinase-1 (MMP1) and increased the level of phosphorylation of NF-κB (p-NF-κB) and Erk (p-Erk) in RA-FLSs. These results suggest that PMPs promote RA-FLSs adhesion and motility presumably by increasing MMP1 via activating Erk-mediated NF-κB pathway.

## Introduction

Rheumatoid arthritis (RA), a chronic autoimmune disease, is characterized by synovial hyperplasia, inflammatory cells infiltration and pannus formation, which erode cartilage and bone, ultimately trigger joint destruction and functional disability [1]. Although detailed pathogenesis of RA remains unclear, accumulating evidences suggest that rheumatoid arthritis fibroblast-like synoviocytes (RA-FLSs) play a crucial role in the pathogenesis of RA [2,3]. It have been reported that activated RA-FLSs predominantly located in synovial intimal lining manifest features such as abnormal proliferation, apoptosis resistance, as well as invasion to adjacent tissues [4,5], and RA-FLSs not only secrete a variety of inflammatory cytokines and chemokines that are responsible for the synovial inflammation processes, but also release some matrix degrading enzymes such as matrix metalloproteinases (MMPs) that aggravate cartilage and bone damage [6–8]. Considering RA-FLSs are able to migrate and invade to normal tissues and accelerate the development of the disease [9,10], inhibition of motility of RA-FLSs might be considered as a potential target for novel treatment strategies for RA.

Platelet-derived microparticles (PMPs), membrane vesicles released during platelet activation [11], were regarded as important inflammatory bodies involving many kinds of bioactive substances, some of which were inflammatory mediators that act on target cells, and were able to amplify intercellular signaling cascade and participate in the inflammatory process [12–14]. Meanwhile, the level of PMPs significantly increased in peripheral blood and synovial fluid of patients with active RA [15]. These findings suggest that PMPs are likely to be involved in the occurrence and development of RA.

Previously, we have demonstrated that platelet-rich plasma can effectively promote migration and invasion of RA-FLSs [16]. In the present study, we further investigated the modulation of PMPs on the adhesion, migration and invasion of RA-FLSs and explored their underlying mechanism.

## Materials and methods

### Cell culture

Human rheumatoid arthritis fibroblast-like synoviocytes (RA-FLSs), purchased from Guangzhou Jennio Biotech Company (Guangzhou, China), were maintained in Dulbecco’s modified Eagle’s medium (DMEM) (Gibco, USA) containing 15% fetal bovine serum (FBS), 100 U/ml penicillin and 100 μg/ml streptomycin in an incubator at 37°C with 5% CO_2_.

### PMPs preparation

PMPs, harvested from patelet-rich plasma (PRP) obtained from Red Cross Blood Station of Yangzhou City (Yangzhou, China). PRP was firstly centrifuged at 3000 rpm for 5 min at 4°C and the sediment was resuspended in Tyrode's buffer. The activation of platelet were performed in 1 mM CaCl_2_, 2 mM MgCl_2_ and 1 mM ADP for 30 min at 37°C. After removal of the platelet aggregates at 4600rpm for 30 min, PMPs were obtained by centrifugation at 13,000 rpm for 1 h at 4°C, and gently washed three times with PBS. Verification of PMPs was assessed by stained with PE-labeled anti-CD41 followed by flow cytometric analysis (FACS CantoII, Becton-Dickinson, USA) and BCA method was used to quantify relative concentration of PMPs.

### Cell adhesion assay

Cell adhesion assay was performed on collagen I, fibronectin or matrigel (BD Biosciences, USA) respectively. For collagen I- or fibronectin-coated, 96-well plates were pre-coated with 10 μg/ml collagen I or 10 μg/ml fibronectin at 4°C overnight. For matrigel-coated, 96-well plates were pre-coated with 200 μg/ml matrigel and incubated at 37°C for 1 h. After blocking with 1% bovine serum albumin for 1 h and washing with PBS, RA-FLSs (2.5×10^5^ cells /ml) incubated with (25, 50 μg/ml) or without PMPs for 48 h were seeded to each well and incubated at 37°C for 45 min. Unattached cells were gently removed and attached cells were quantified by CCK-8 (Obio Technology, Shanghai, China) stained, then absorbance was measured at 450 nm. The independent assays were performed for four times.

### Cell migration and invasion assay

Cell migration was examined using the wound healing assay and transwell assay. For wound healing assay, 1×10^5^ cells per well were seeded into 24-well plates, and incubated in DMEM with 15% FBS overnight. After treated with different concentrations of PMPs (0, 25, 50 μg/ml) in DMEM with 15% FBS for 12 h and then starved in serum-free DMEM for 12 h, the cell monolayers were scratched with a 200-μl pipette tip to create a wound. Then cells migrating into the wound area was monitored by capturing photographs at indicated time. The assays were repeated three times. For the transwell migration assay, cells were starved off in serum-free DMEM with various concentration of PMPs (0, 25, 50 μg/ml) for 12 h, and 2×10^4^ cells were seeded into the upper tranwell chambers (pore size = 8.0 μm; Corning, USA) of the 24-well transwell plates. Then DMEM with 15% FBS and corresponding PMPs were added into the lower chambers. After incubation at 37°C for 24 h, the cells on the upper surface of the chamber membrane were gently wiped with a cotton swab, and the cells migrated to the lower surface were fixed, stained, and then counted in eight random microscopic fields (100×). The independent assays were repeated four times. Transwell invasion assay were mainly consistent with transwell migration assay, and the only difference was that 100 μl matrigel (3.75 mg/ml, BD Biosciences, USA) was added into the chambers previously overnight and then the basement membranes were fully hydrated with DMEM for 1 h at 37°C. Four independent experiments were performed.

### Microarray analysis

Microarray analysis was performed to evaluate gene expression in RA-FLSs after treated with (50 μg/ml) or without PMPs (0 μg/ml) for 48 h. Total RNA was extracted using Trizol reagent (Vazyme, Nanjing, China) according to the manufacturer’s instructions. RNA quality and concentration was determined by NanoDrop 2000 and Agilent Bioanalyzer 2100. In this study, RNA samples with 1.8<A260/A280<2.0, RIN (RNA Integrity Number)> = 7.0 and 28s/18s>0.7 were considered satisfactory and used for subsequent analysis, which were detected by Affymetrix GeneChip system (Genechem Co., Ltd, Shanghai, China).

### RT-qPCR

Total RNA was extracted in the same way as for the microarray analysis and cDNA was synthesized using the Hiscript First-strand cDNA Synthesis Kit (Vazyme, Nanjing, China) according to the manufacturer’s instructions. qPCR assay was performed using ABI 7500 Real-Time PCR System (Applied Biosystems, American). Primers used in this study were as follows: MMP1 forward, 5′-GGGAGATCATCGGGACAACTC-3′, and reverse, 5′-GGGCCTGGTTGAAAAGCA T-3′; MMP2 forward 5′-CCGTCGCCCAT CATCAAGTT-3′, and reverse, 5′-CTGTCTGGGGCAGTCCAAAG-3′; GAPDH forward, 5′-GCACCGTCAAGGCTGAGAAC-3′, and reverse, 5′-TGGTGAAGACGCC AGTGGA-3′. The relative levels of the target mRNA normalized to GAPDH mRNA were evaluated by 2^-ΔΔC^ method.

### Western blot

Cell lysates were prepared with a total protein extraction kit (Vazyme, Nanjing, China) and protein concentrations were quantified with BCA method. The protein samples were separated on sodium dodecy sulfate-polyacrylamide gel electrophoresis (SDS-PAGE) and transferred to polyvinylidene fluoride membranes (Millipore, USA). After blocking with 5% nonfat dried milk, the membranes were incubated with monoclonal antibodies at 1: 1000 (MMP1, MMP2, IκB, p-IκB, NF-κB, p-NF-κB, Erk, p-Erk, Akt, p-Akt and GAPDH, Cell Signaling Technology, USA), and followed by incubation with goat anti-mouse or anti-rabbit IgG secondary antibodies (Cell Signaling Technology, USA). The membranes were visualized using Pierce ECL Plus Western Blotting substrate (Thermo Scientific, USA) and analyzed by Alphaview software. GAPDH was used for loading control. All experiments were conducted in triplicate.

### Statistical analysis

All results are described as the mean ± standard deviations. Statistical differences were analyzed using SPSS 22.0 software and the value of *p* < 0.05 was considered statistically significant.

## Results

### 1. PMPs increase the adhesion of RA-FLSs to extracellular matrix (ECM)

**Fig 1:**
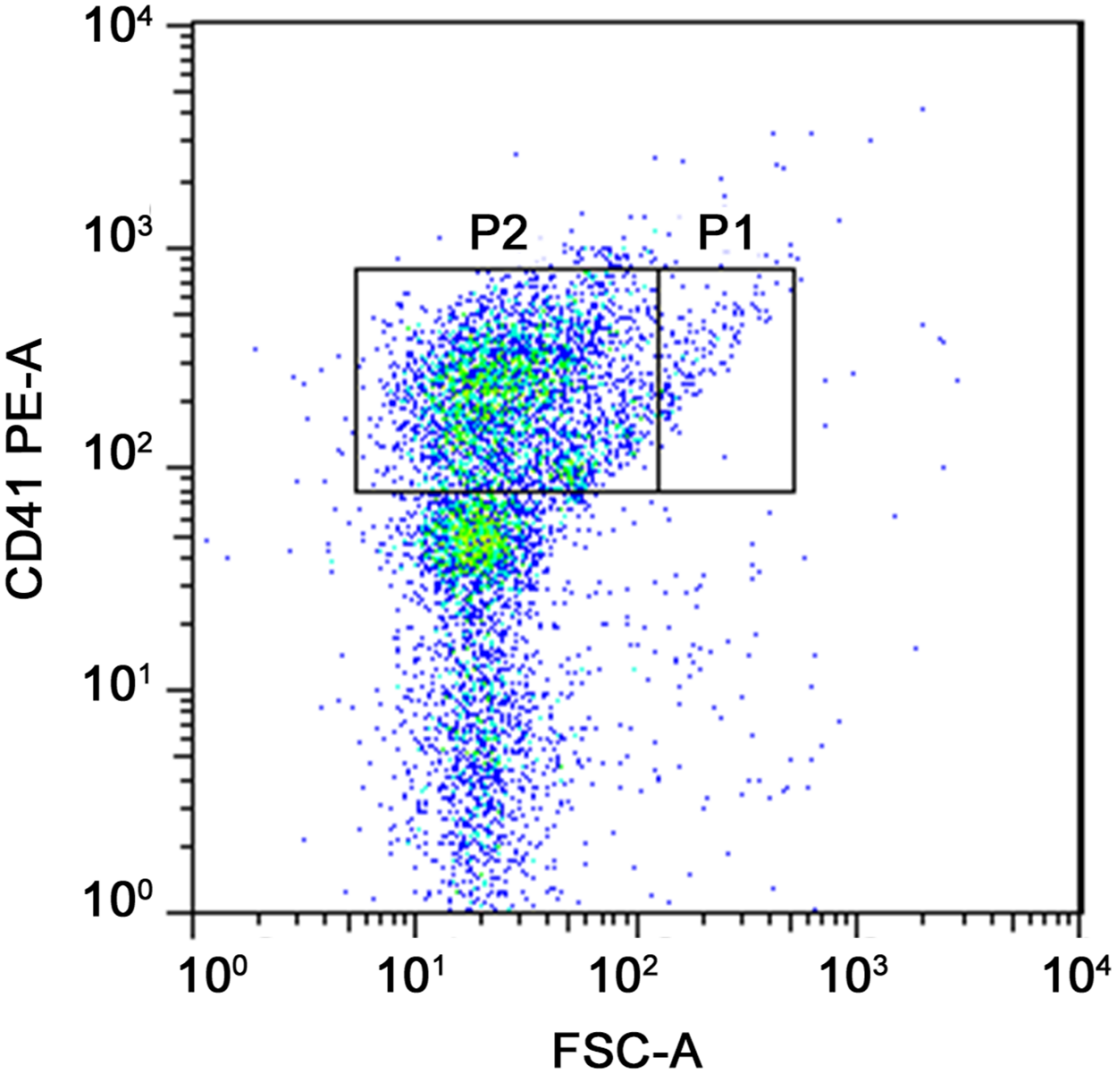
Verification of PMPs by flow cytometry. PMPs were isolated from platelet-rich plasma and stained with PE-labeled anti-CD41.The events in P2 were PMPs, which were compared to 0.82 μm standard microspheres.

In order to study the influence of PMPs on adhesion and motility of RA-FLSs, PMPs were firstly prepared from platelet-rich plasma and verified by flow cytometric analysis, which were stained with PE-labeled anti-CD41 and compared to 0.82 μm standard microspheres (Fig 1).

**Fig 2:**
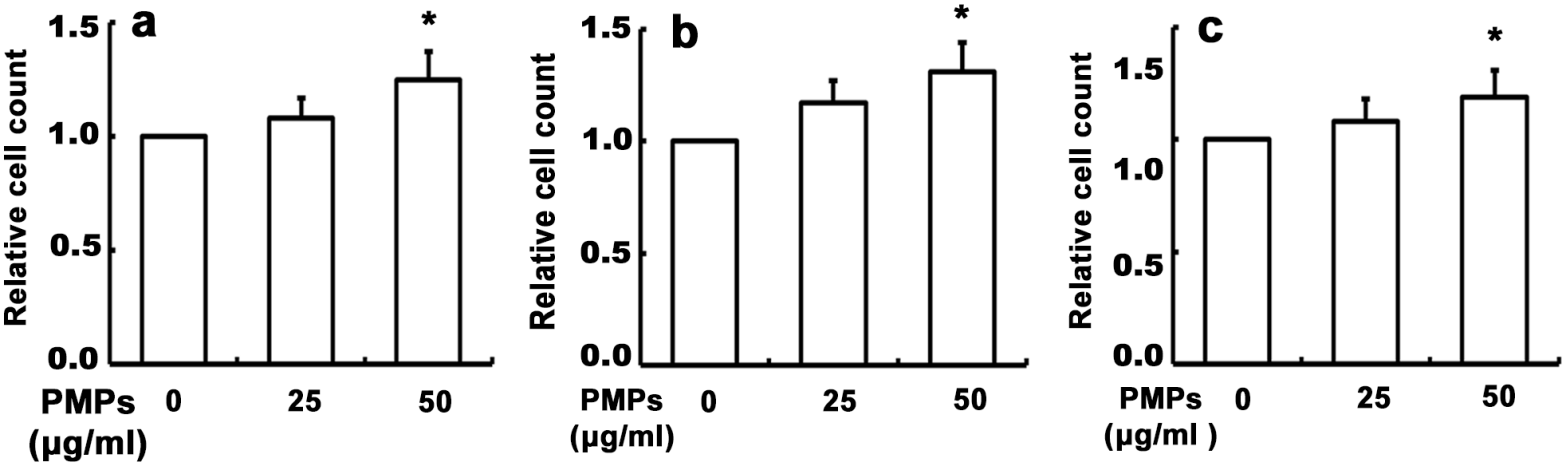
Effects of PMPs on adhesion of RA-FLSs. RA-FLSs were seeded onto the collagen I- (a), fibronectin- (b) or matrigel- (c) coated 96-well culture plates, respectively, and incubated at 37°C for 45 min. The CCK-8 assays were conducted to quantify the number of the adhesive cells by absorbance at 450 nm, and performed in six wells from four independent experiments. (*p < 0.05 vs. 0 μg/ml PMPs).

Since cell adhesion to ECM and shedding from ECM are considered to be initial steps in cellular migration and invasion [17], we firstly examined whether PMPs influence adhesion of RA-FLSs to ECM. After treatment with different concentration of PMPs for 24 h, the cells were seeded on collagen I-, fibronectin- or matrigel-coated 96-well culture plates, respectively, and incubated at 37°C for 45 min. As shown in Fig 2, PMPs promoted the adhesion of RA-FLSs onto collagen I, fibronectin and matrigel, the statistically significance was shown in the group of 50 μg/ml PMPs, indicating that PMPs might play a positive regulatory role in the adhesion of RA-FLSs to ECM.

### 2. PMPs promote the migration and invasion of RA-FLSs

**Fig 3:**
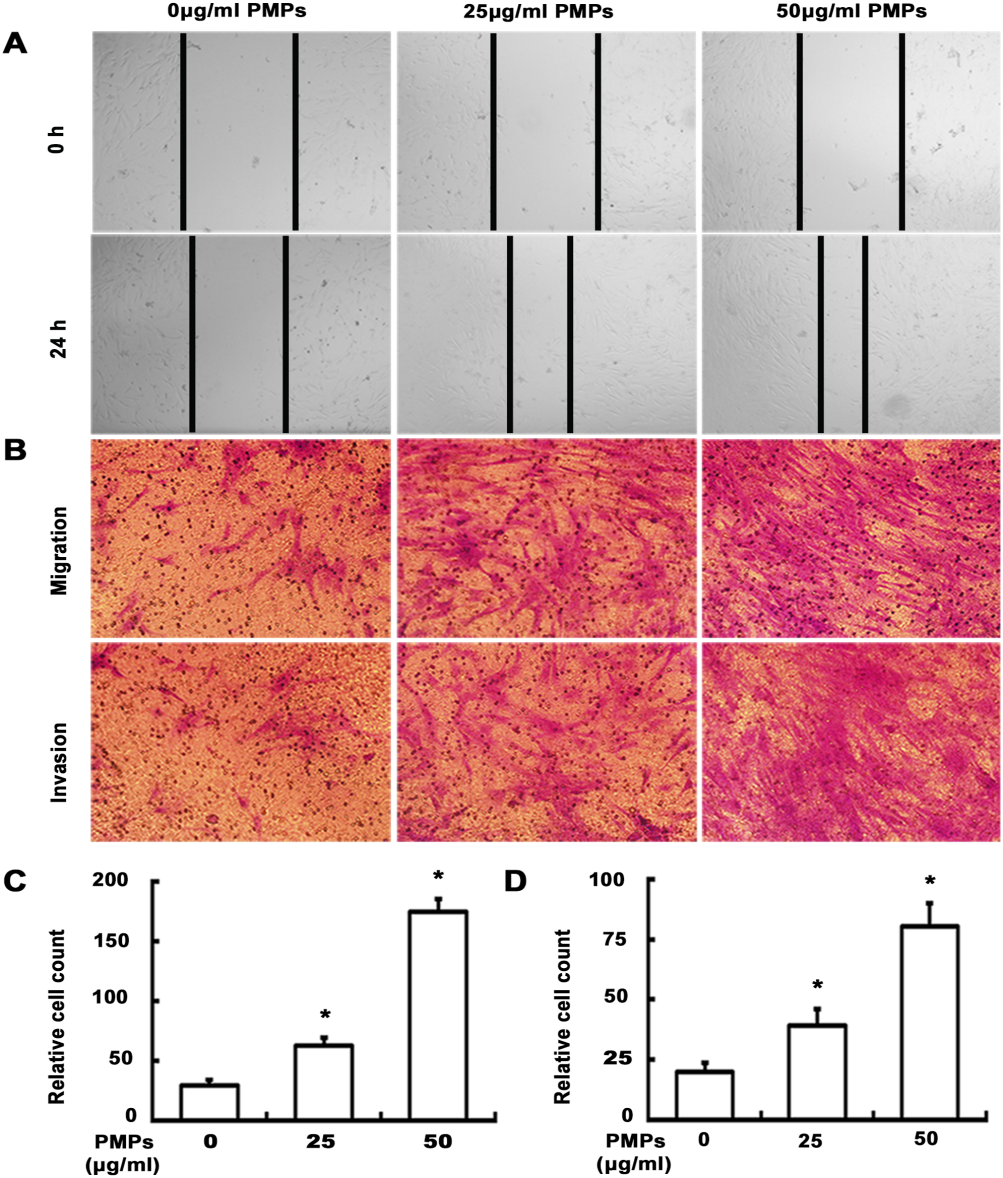
Effect of PMPs on migration and invasion of RA-FLSs. (A) The wound healing assay were performed after RA-FLSs treated with different concentrations of PMPs for 24 h. (B) Transwell migration and invasion assays were performed after RA-FLSs treated with PMPs for 24 h. (C and D) Quantification of the migration (C) and invasion (D) was shown. (*p < 0.05 vs. 0 μg/ml PMPs).

To evaluate the effect of PMPs on the migratory capacity of RA-FLSs, we next conducted a wound healing assay. Results from Fig 3A demonstrated that RA-FLSs incubated with PMPs for 24 h exhibited faster closure of the wound area as compared to the group of 0 μg/ml PMPs. As expected, transwell migration assay also indicated that PMPs enhanced the migration of RA-FLSs. Furthermore, the similar effect of PMPs on cellular invasion was observed in transwell invasion assay (Fig 3B–3D). Taken together, our observations strongly suggest that PMPs play an important role in promoting the migratory and invasive behaviors of RA-FLSs.

### 3. Gene expression analysis

**Fig 4:**
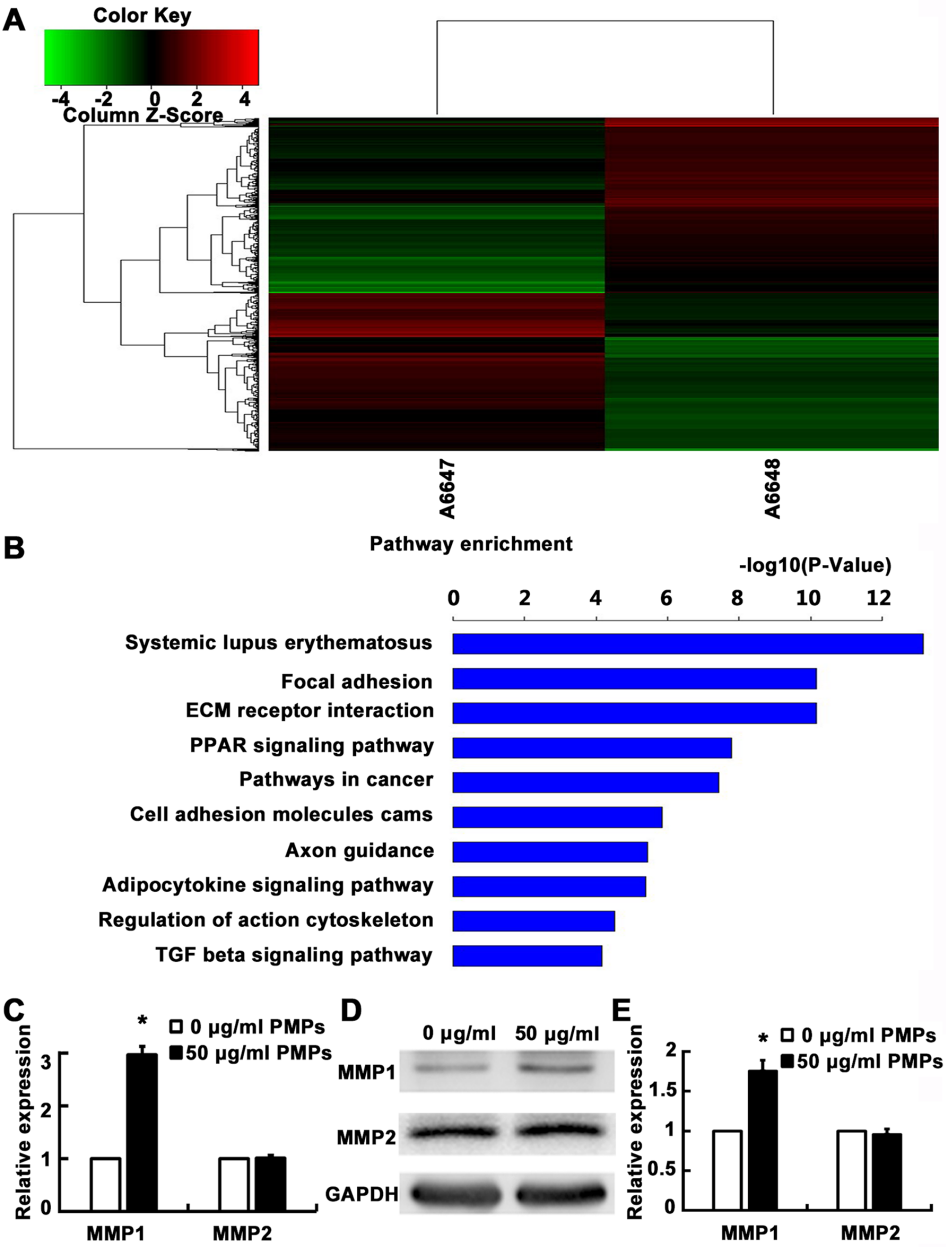
Effects of PMPs on gene expression of RA-FLSs. (A) The clustering analysis compared the differences of gene expression between the group without (A6647) and with 50 μg/ml (A6648) PMPs: Red region, genes up-regulated in RA-FLSs. Green region, genes down-regulated in RA-FLSs. (B) Top 10 differential genes of pathway enrichment analysis are listed. (C) Relative mRNA levels of MMP1 and MMP2 of RA-FLSs after treated with PMPs were measured by RT-qPCR and GAPDH was used as an equal loading control. (*p < 0.05 vs. 0 μg/ml PMPs). (D) Protein levels of MMP1 and MMP2 were detected by western blot and normalized to GAPDH. (E) Quantification of protein levels was shown (*p < 0.05 vs. 0 μg/ml PMPs).

To explore the possible mechanism of PMPs promoting RA-FLSs adhesion and motility, we performed microarray analysis to evaluate the effect of PMPs on gene expression of RA-FLSs. Total RNA was extracted from RA-FLSs after incubation with PMPs (50 μg/ml) or without PMPs (0 μg/ml) for 48 h and subjected to Affymetrix microarray analysis. As shown in Fig 4A, compared with the group of 0 μg/ml PMPs, up-regulation of 777 genes and down-regulation of 702 genes were shown in group of 50 μg/ml PMPs. The further pathway enrichment analysis demonstrated that differentially expressed genes mainly focused on ECM receptor interactions, focal adhesion and cell adhesion molecules (Fig 4B). We also found that the expression of MMP1 was markedly up-regulated, while other MMPs, such as MMP2, MMP9, had no obvious alteration. Naturally, we performed reverse transcription quantitative real-time PCR (RT-qPCR) to validate whether PMPs alter the expression of MMP1 and MMP2. As expected, the results were consistent with microarray data (Fig 4C). The further western blot analysis also revealed that MMP1 rather than MMP2 were increased accordingly after the treatment of PMPs (Fig 4D and 4E). These findings suggested MMP1 might be at least in part involved in the regulation of motility of RA-FLSs.

### 4. PMPs activate NF-κB pathway and increase the expression of p-Erk

**Fig 5:**
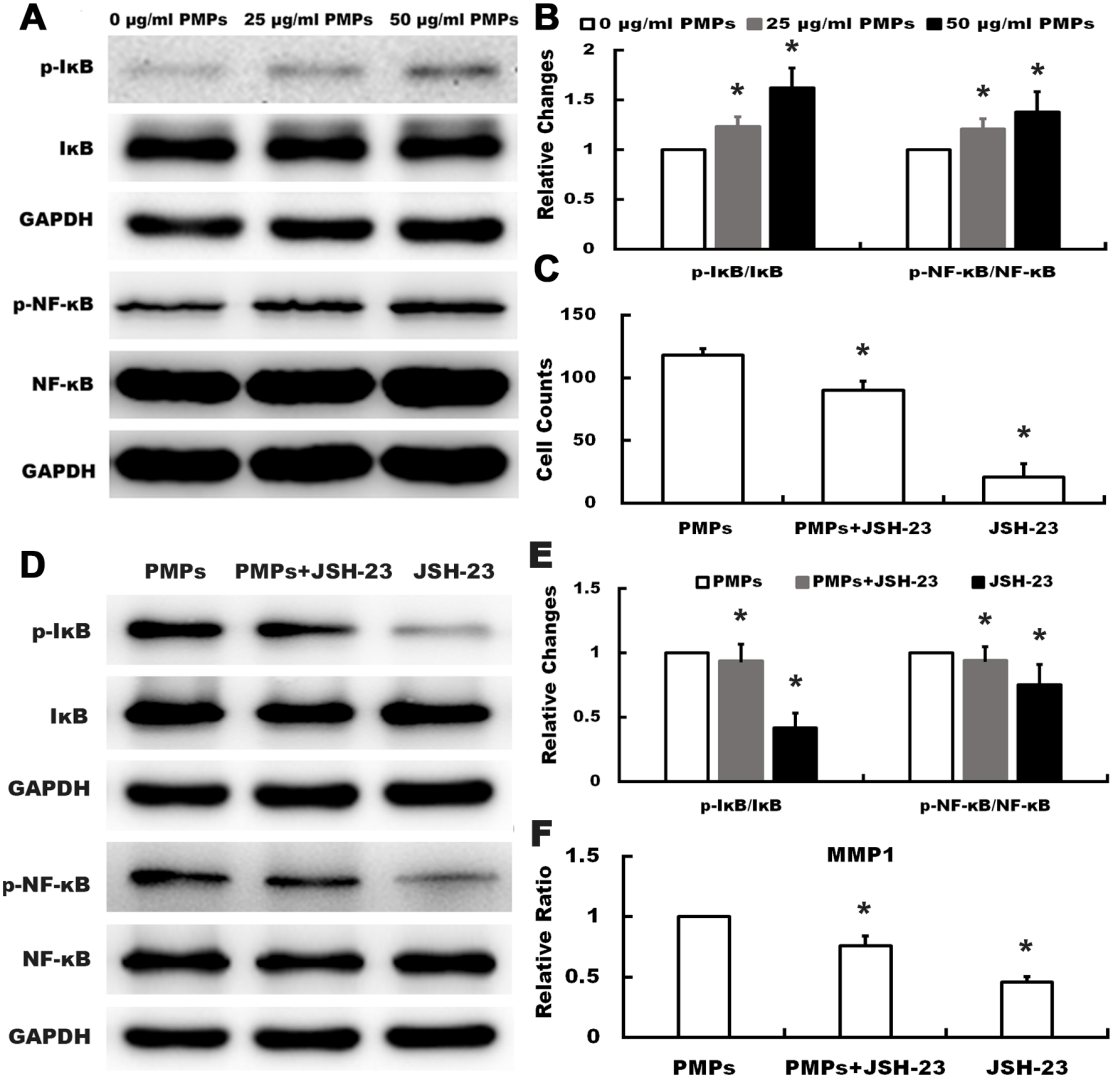
Effects of PMPs on the expression and activation of IκB and NF-κB in RA-FLSs. (A and B) Western blotting was performed to detect the expression of p-IκB, IκB, NF-κB and p-NF-κB of RA-FLSs after treated with PMPs. The quantification was expressed as fold-change of 0μg/ml PMPs. (*p < 0.05 vs. 0 μg/ml PMPs) (C) Quantification of the migration assay of RA-FLSs treated with PMPs and JSH-23 was shown. (*p < 0.05 vs. PMPs) (D and E) After treated with PMPs and JSH-23, the expression of p-IκB, IκB, p-NF-κB and NF-κB of RA-FLSs were detected by western blot. (*p < 0.05 vs. PMPs) (F) After treated with PMPs and JSH-23, the expression of MMP1 was detected by RT-qPCR. (*p < 0.05 vs. PMPs).

Since expression of MMP1 was regulated by activation of NF-κB signaling in many cell types [18,19], we conducted western blot analysis to investigate the effect of PMPs on the expression and activation of NF-κB signaling molecules in RA-FLSs. As shown in Fig 5A and 5B, when compared with the group of 0 μg/ml PMPs, PMPs markedly increased the phosphorylation of IκB and NF-κB, whereas there was no significant alteration in the expression level of IκB or NF-κB. To further verify PMPs indeed affected MMP1 by activating NF-κB signaling, JSH-23 (20 μM, Selleckchem, USA), the NF-κB specific inhibitor, was selected for subsequent analysis. The results of the transwell assay showed that JSH-23 could significantly inhibit migration of RA-FLSs stimulated by PMPs (Fig 5C). Meanwhile, data from Fig 5D–5F demonstrated that JSH-23 decreased the levels of p-IκB, p-NF-κB and MMP1. Taken together, these results indicated that the expression of MMP1 in RA-FLSs induced by PMPs possibly through activating NF-κB signaling.

**Fig 6:**
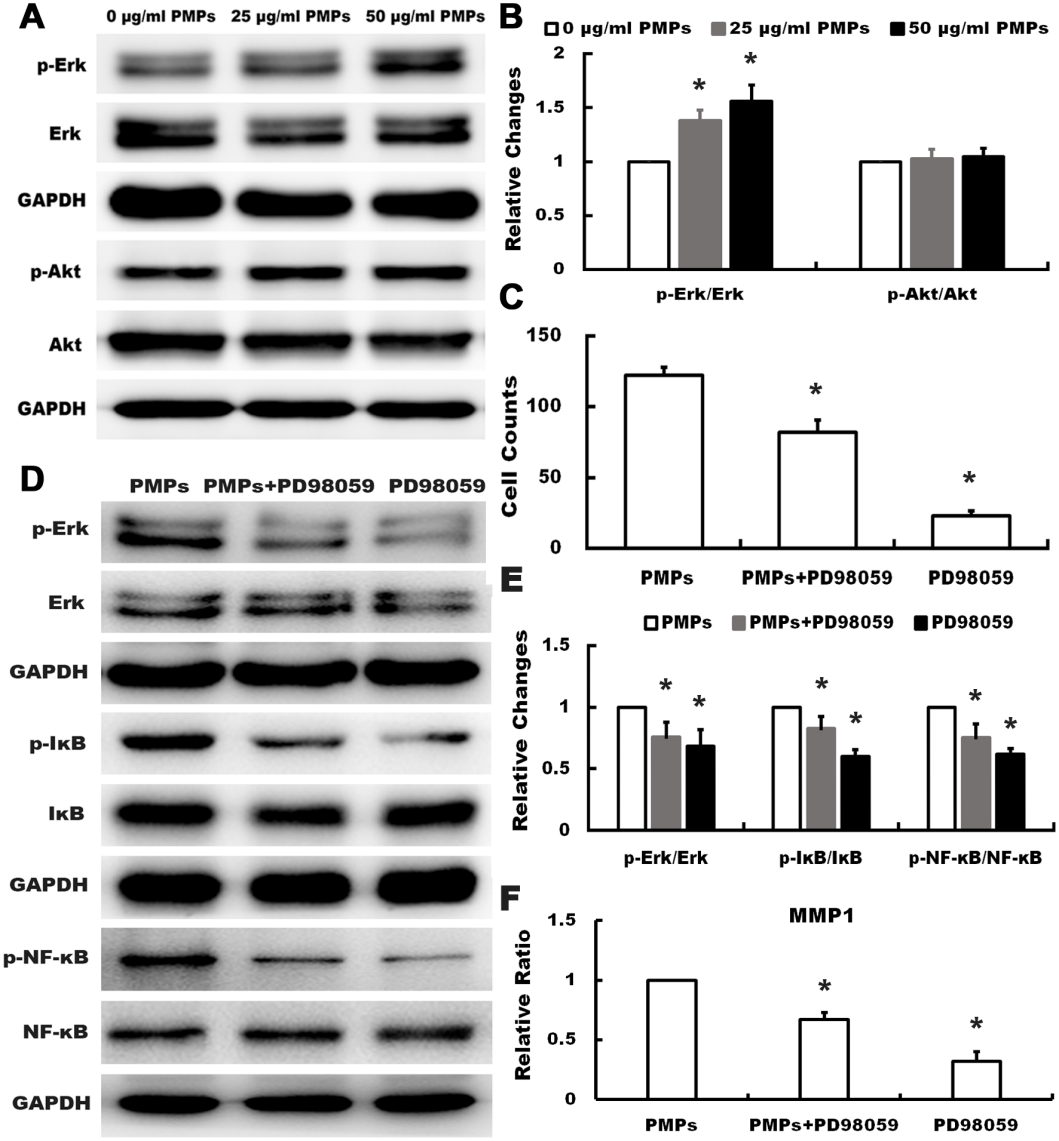
Effects of PMPs on the expression and activation of Erk and Akt in RA-FLSs. (A and B) Protein levels of p-Erk, Erk, Akt and p-Akt were analyzed from PMPs-treated RA-FLSs with corresponding antibodies. Quantification analysis was shown and expressed as fold-change. (*p < 0.05 vs. 0 μg/ml PMPs) (C) Quantification of the migration assay of RA-FLSs with PMPs and PD98059 was shown. (*p < 0.05 vs. PMPs) (D and E) Western blotting was performed to detect Erk, p-Erk, IκB, p- IκB, NF-κB and p- NF-κB of RA-FLSs after treated with PMPs and PD98059. (*p < 0.05 vs. PMPs) (F)RT-qPCR was conducted to detect the expression of MMP1 in RA-FLSs after treated with PMPs and PD98059. (*p < 0.05 vs. PMPs).

Considering both Erk and Akt activation are common signal pathways for activation of NF-κB signaling [20,21], we further detected the expression and activation of Erk and Akt in RA-FLSs in response to PMPs. Data from Fig 6A and 6B shown that PMPs promoted phosphorylation of Erk but not Akt, and no significant alteration of Erk and Akt were observed in three groups. To prove the expression of MMP1 was altered by PMPs through Erk-NF-κB signaling pathway, we treated the cells with PD98059 (25 μM, Cell Signaling Technology, USA) which was the Erk specific inhibitor. As shown in Fig 6C, transwell migration assay proved that PD98059 could inhibit migration of RA-FLSs, and data from Fig 6D–6F showed PD98059 suppressed the expression of p-Erk, p-IκB, p-NF-κB and MMP1 induced by PMPs. All the results suggested that PMPs might up-regulate MMP1 in RA-FLSs presumably through Erk activating NF-κB signaling.

## Discussion

Accumulating evidences showed that activation of platelet play a crucial role in many autoimmune diseases, such as RA, systemic lupus erythematosus and ankylosing spondylitis, and the process in platelet activation was accompanied by the formation of PMPs, which amplify the inflammatory reaction of autoimmune disease as a kind of inflammatory body [13,22,23]. In addition, it have been documented that the levels of PMPs both increased in peripheral blood and synovial fluid of RA patients [15], and PMPs might be associated with disease activity of RA and involved in the pathogenesis of RA. In the present study, we revealed that PMPs play a positive role in regulation of adhesion and motility of RA-FLSs presumably through Erk activating NF-κB signaling.

Since RA-FLSs have the properties of migrating from joint to joint, as well as attaching to and invading into cartilage and bone [9,24], we firstly employed wound healing, transwell migration and transwell invasion assays to evaluate the effect of PMPs on the motility of RA-FLSs. Results showed that PMPs obviously promoted the migration and invasion of RA-FLSs. Meanwhile, cell adhesion assay also showed that PMPs increased the adhesion to ECM (collagen I, fibronectin and matrigel), which is the precondition of cell motility. In order to explore the underlying mechanism, we performed gene microarray analysis, data showed that 1479 genes were differentially expressed (777 genes up-regulated and 702 genes down-regulated), further enrichment analysis indicated that MMP1 expression obviously up-regulated, whereas other MMPs expression had no obvious alteration. It is well known that MMPs family are vital enzymes in bone and cartilage injury of patients with RA and may promote the RA-FLSs to break through the basement membrane to degrade ECM [25,26], thereby, we used RT-qPCR and western blot to verify the alteration in gene expression of RA-FLSs after treatment with PMPs, and the results were consistent with microarray analysis. Since up-regulation of MMP1 might result from activating NF-κB signaling in many cell types like tumor cells and mesenchymal stem cells [18,19], we found that PMPs markedly increased the p-IκB and p-NF-κB, manifesting the positive effect of PMPs on activating NF-κB signaling in RA-FLSs. Meanwhile, on the basic of PMPs, we selected NF-κB specific inhibitor (JSH-23) to treat RA-FLSs, and the results suggested that not only the migration Migration capacity was limited, but also the levels of p-IκB, p-NF-κB and MMP1 were decreased, indicating that the alteration of MMP1 was induced by PMPs possibly through activating NF-κB signaling. It has been proved that activated NF-κB signaling is modulated by the activation of Erk or Akt in other cells, while our results further revealed that PMPs increased the phosphorylation of Erk, but not Akt. Moreover, Erk specific inhibitor (PD98059) was taken to verify the results. We found that PD98059 could inhibit cell migration ability and the expression of p-Erk, p-IκB, p-NF-κB and MMP1 induce by PMPs. These results suggest that PMPs promote the adhesion and motility of RA-FLSs presumably by up-regulating MMP1 expression through Erk activating NF-κB signaling.

In summary, PMPs promoted RA-FLSs adhesion onto ECM, migration and invasion of RA-FLSs. The underlying mechanism may be the increased expression of MMP1 by activation of NF-κB pathway, which is activated by Erk phosphorylation. Further study of the underlying mechanism as to how PMPs coordinate the Erk-NF-κB-MMP1 signaling is under way, however, the findings present here may reinforce a role for PMPs as a novel molecular target for RA-FLSs.
